# Prognostic value of immune checkpoint molecules in head and neck cancer: a meta-analysis

**DOI:** 10.18632/aging.101756

**Published:** 2019-01-22

**Authors:** Yi-Qun Jia, Bo Yang, Li-Ling Wen, Wen-Xin Mu, Zhi Wang, Bin Cheng

**Affiliations:** 1 Guangdong Provincial Key Laboratory of Stomatology, Guanghua School of Stomatology, Hospital of Stomatology, Sun Yat-sen University, Guangzhou, Guangdong 510055, China; * Equal contribution

**Keywords:** immune checkpoint molecule, prognosis, survival, head and neck cancer, meta-analysis

## Abstract

Immune checkpoint molecules are important targets in cancer immunotherapy, but their association with prognosis in patients with head and neck cancer is controversial. In this meta-analysis, we searched for 12 immune checkpoint molecules in the PubMed, Embase and Cochrane Library databases and retrieved 52 studies with 7127 participants. Among the molecules included in the search, indoleamine 2, 3-dioxygenase (IDO), programmed death ligand 1 (PD-L1), and programmed death 1 (PD-1) met the inclusion criteria for further analysis. Higher expression of IDO was associated with poorer overall survival in head and neck cancer patients (*P* = 0.011), but higher expression of PD-L1 correlated with better overall survival specifically in nasopharyngeal carcinoma patients (*P* = 0.01). In a sensitivity analysis, higher PD-L1 expression correlated with better progression-free survival (*P* = 0.043), and was associated with better overall survival in Caucasian subjects (*P* = 0.02), nasopharyngeal carcinoma patients (*P* = 0.015), and studies with small sample sizes (*P* = 0.001). PD-1 had no prognostic significance. There was no publication bias affecting the results. Thus, among the immune checkpoint molecules, IDO and PD-L1 are potential prognostic predictors in head and neck cancer.

## Introduction

Head and neck cancer (HNC) is the sixth most common malignancy worldwide [1]. Most patients exhibit advanced-stage disease, including regional lymph node involvement, and 10% of patients have distant metastases [2]. The traditional treatment options for HNC are surgery, radiotherapy and chemotherapy [3], which have severe adverse effects. Furthermore, some patients do not benefit much from these treatments, and are likely to relapse. Anatomic complexities often lead to malfunctions in speaking, swallowing and breathing after treatments, hampering patients’ long-term quality of life [4]. Although there have been certain advances in treatment, the overall survival of HNC patients is still unsatisfactory, and the five-year survival rate is less than 50% [5–7].

Immunosuppressive patients are prone to suffer from HNC [8], although the predominant causes of HNC are tobacco and alcohol consumption [4] and viral infections [9,10]. Among the functions of the immune cells, immune checkpoint activity has been reported to be involved in the surveillance of tumor development and progression [11]. Immune checkpoint molecules including programmed death 1 (PD-1) [12,13], indoleamine 2, 3-dioxygenase (IDO) [14,15], B7-H3 [16,17], lymphocyte activation gene 3 (LAG-3) [18], cytotoxic T-lymphocyte-associated protein 4 (CTLA-4) [19], programmed death ligand 2 (PD-L2) [20], V-domain Ig suppressor of T cell activation (VISTA) [21], B7-H4 [22] and programmed death ligand 1 (PD-L1) [23–25] have been used as markers to evaluate the prognosis of HNC. However, the survival rates of patients with high expression of immune checkpoint molecules have differed according to the overexpressed molecule.

In the present study, we performed a systematic review of the available literature on this topic in PubMed, Embase and the Cochrane Library. Then, we conducted a meta-analysis of the survival rates (including overall survival [OS], disease-free survival [DFS], progression-free survival [PFS], disease-specific survival [DSS] and distant metastases-free survival [DMFS]) of patients expressing different levels of immune checkpoint molecules.

## RESULTS

### Study characteristics

**Figure 1:**
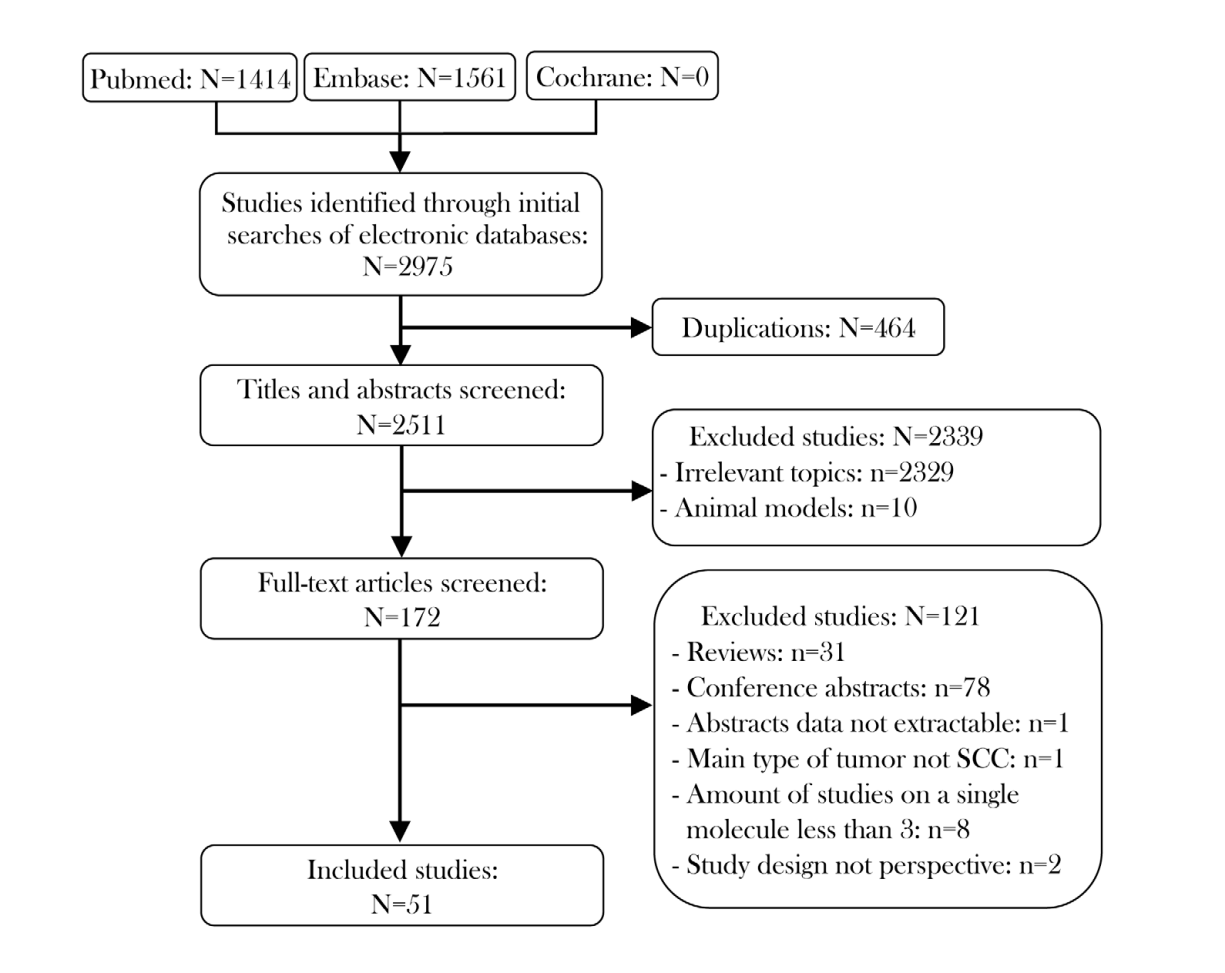
Flow diagram of studies identified, included and excluded.

The characteristics of the included studies are shown in Table 1. There were 52 prospective studies comparing contemporary series of patients (level of evidence: 3b) in 51 articles. These studies included 7127 patients and met the criteria for meta-analysis. The literature selection procedure is presented in Figure 1. The included articles were evaluated by the Newcastle–Ottawa Scale (NOS; Supplementary Table 1), and all the articles were published between 2010 and 2018. Roughly half of the studies were conducted in Asia (n=23), while the remainder were conducted in Europe (n=18), North and South America (n=6), Oceania (n=4) and Africa (n=1). Thus, the samples included in this meta-analysis covered most of the continents of the world. In terms of the immune checkpoint molecules, the majority of the studies evaluated PD-L1 (n=40), while the rest assessed PD-1 (n=8) and IDO (n=4). The sample sizes of the included studies ranged from 38 to 517. With reference to the mean value of all the samples, 17 studies were considered to have a large sample size (n > 139), while 35 had a small sample size (n ≤ 139). Forty-three studies explored the prognostic value of their chosen immune checkpoint molecule for OS, 19 for DFS, 6 for PFS, 5 for DSS and 3 for DMFS.

**Table 1:** Characteristics of included studies.

Author and year	Target	Country / Region	Ethnicity	Tumor location	Sample size	Gender M/F	Cut-off value	Detection method	TNM stage	Outcome	HR estimation	Study design	NOS score
Ahn et al. 2017 [23]	PD-L1	Korea	Asian	OSCC	68	45/23	Grade > 1	IHC	I-IV	OS DFS	reported	P	7
Badoual et al. 2013 [12]	PD-1	France	Caucasian	HNSCC	64	NA	> median	IF	I-IV	OS	reported	P	6
Balempas et al. 2017 [36]	PD-L1	Germany	Caucasian	HNSCC	161	131/30	> 5%	IHC	I-IV	OS DMFS	reported	P	7
Ben-Haj-Ayed et al. 2016 [14]	IDO	Tunisia	Caucasian	NPC	71	48/23	> median	IHC	I-IV	OS DFS	reported	P	7
Birtalan et al. 2017 [24]	PD-L1	Hungary	Caucasian	HNSCC	106	90/16	Score > 0%	IHC	I-IV	DSS	reported	P	6
Budczies et al. 2016 [25]	PD-L1	Germany	Caucasian	HNSCC	517	NA	> median	qRT-PCR	NA	OS DFS	reported	P	5
Chan et al. 2017 [46]	PD-L1	USA	Caucasian	NPC	161	117/44	≥ 1%	IHC	I-IV	OS PFS	reported	P	6
Chang et al. 2017 [47]	PD-L1	Philippines	Asian	NPC	56	43/13	> 1%	IHC	I-IV	OS	reported	P	5
Chen et al. 2015 [48]	PD-L1	Taiwan	Asian	OSCC	218	145/73	> 5%	IHC	I-IV	OS	reported	P	7
Chen et al. 2017 [49]	PD-L1	China	Asian	HNSCC	496	NA	> 5%	qRT-PCR	I-IV	OS	reported	P	7
Cho et al. 2011 [50]	PD-L1	Korea	Asian	OSCC	45	32/13	Grade > 1	IHC	I-IV	OS	estimated	P	6
De Meulenaere et al. 2017 [51]	PD-L1	Belgium	Caucasian	OSCC	99	82/17	> 1%	IHC	I-IV	OS DFS	reported	P	6
Fang et al. 2014 [52]	PD-L1	China	Asian	NPC	139	113/26	> 35%	IHC	I-IV	DFS	estimated	P	6
Feng et al. 2017 [53]	PD-L1	USA	Caucasian	OSCC	119	74/45	< 30 μm	IHC	I-IV	OS	estimated	P	6
Fiedler et al. 2018 [54]	PD-L1	Germany	Caucasian	HNSCC	82	73/9	> 5%	IHC	I-IV	OS	reported	P	7
Hanna et al. 2018 [37]	PD-L1	USA	Caucasian	OSCC	81	49/32	> 10%	IHC	I-IV	OS	reported	P	7
Hong et al. 2016 [55]	PD-L1	Australia	Caucasian	OSCC	99	79/20	> 25%	IHC	I-IV	OS	reported	P	6
Hsu et al. 2010 [13]	PD-1	Taiwan	Asian	NPC	46	39/7	> median	IHC	NA	OS DFS	reported	P	4
Kansy et al. 2017 [56]	PD-1	Germany	Caucasian	HNSCC	56	NA	NA	FACS	I-IV	DFS	reported	P	6
Kim et al. 2016 [57]	PD-1	Korea	Asian	HNSCC	402	302/100	> 5%	IHC	I-IV	OS DFS	reported	P	6
Kim et al. 2016 [58]	PD-1	Korea	Asian	OSCC	133	120/13	> 5%	IHC	I-IV	OS	reported	P	7
Kogashiwa et al. 2017 [35]	PD-L1	Japan	Asian	OSCC	84	57/27	> 5%	IHC	I-IV	OS PFS	reported	P	7
Laimer et al. 2011 [15]	IDO	Austria	Caucasian	OSCC	88	67/21	> 4	IHC	I-IV	OS	reported	P	7
Larbcharoensub et al. 2018 [59]	PD-L1	Thailand	Asian	NPC	114	77/67	≥ 5%	IHC	I-IV	OS	estimated	P	7
Lee et al. 2016 [60]	PD-L1	Hong Kong	Asian	NPC	104	85/19	> 1	IHC	I-IV	PFS DMFS OS	reported	P	5
Li et al. 2017 [61]	PD-L1	China	Asian	NPC	62	40/14	> 20%	IHC	I-IV	DFS	reported	P	5
Lin et al. 2015 [30]	PD-L1	Taiwan	Asian	OSCC	305	236/69	> 1	IHC	I-IV	OS	reported	P	6
Muller et al. 2017 [62]	PD-L1	Germany	Caucasian	HNSCC	293	82/16 142/53 (224/69)	Score ≥ 1	IHC	I-IV	OS	reported	P	6
Ock et al. 2016 [63]	PD-L1	South Korea	Asian	HNSCC	141	40/10 61/30 (101/40)	≥ 5%	IHC	I-IV	OS	reported	P	6
Oguejiofor et al. 2017 [64]	PD-L1	UK	Caucasian	OPSCC	124	NA	> 5%	IHC	I-IV	OS	reported	P	7
Oliveira-Costa et al. 2015 [65]	PD-L1	Brazil	Caucasian	OSCC	142	125/17	≥ 5%	IHC	I-III	DSS	reported	P	6
Ono et al. 2017 [66]	PD-L1	Japan	Asian	HPSCC	83	79/4	≥ 1%	IHC	III-IV	OS PFS	reported	P	6
Ono et al. 2018 [67]	PD-L1	Japan	Asian	NPC	66	54/12	≥ 5%	IHC	I-IV	OS PFS	reported	P	7
Ou et al. 2017 [68]	PD-L1	France	Caucasian	HNSCC	38	NA	≥ 1%	IHC	III-IV	OS PFS	estimated	P	7
Qu et al. 2018 [69]	PD-L1	China	Asian	NPC	96	72/24	> 10%	IHC	I-IV	DMFS	estimated	P	6
Riobello et al. 2018 [70]	PD-L1	Spain	Caucasian	SSCC	53	37/16	≥ 5%	IHC	I-IV	OS DFS DSS	reported	P	5
Roper et al. 2017 [71]	PD-L1	Australia	Caucasian	HNSCC	74	64/10	> 5%	IHC	NA	DFS	reported	P	6
Satgunaseelan et al. 2016 [72]	PD-L1	Australia	Caucasian	OSCC	217	130/87	Score ≥ 1	IHC	NA	DSS	estimated	P	6
Schneider et al. 2018 [73]	PD-1 PD-L1	Austria	Caucasian	HNSCC	129	97/28	> 5%	IHC	I-IV	OS DFS	reported	P	7
Seppälä et al. 2016 [74]	IDO	Finland	Caucasian	OSCC	58	29/29	> 0	IHC	I-III	OS	reported	P	6
Solomon et al. 2018 [75]	PD-L1	Australia	Caucasian	OSCC	190	157/33	≥ 5%	IHC	I-IV	OS	reported	P	7
Steuer et al. 2018 [76]	PD-1	USA	Caucasian	OPSCC	97	81/16	Score > 1	IHC	I-IV	OS	reported	P	7
Strati et al. 2017 [77]	PD-L1	Greece	Caucasian	HNSCC	113	75/19	NA	qRT-PCR	I-IV	OS PFS	reported	P	5
Straub et al. 2016 [78]	PD-L1	Germany	Caucasian	OSCC	80	54/26	> 5%	IHC FISH	I-IV	OS DFS	estimated	P	7
Tang et al. 2017 [79]	PD-1	China	Asian	NPC	96	NA	NA	IHC	NA	OS	estimated	P	6
Ukpo et al. 2013 [80]	PD-L1	USA	Caucasian	OPSCC	181	162/19	> 5%	IHC	I-IV	OS DFS DSS	reported	P	7
Vassilakopoulou et al. 2016 [81]	PD-L1	Greece	Caucasian	LSCC	260	249/11	> 59th percentile of AQUA score	IHC	I-IV	OS DFS	reported	P	7
Ye et al. 2013 [82]	IDO	China	Asian	LSCC	187	179/8	NA	IHC	I-IV	OS DFS	reported	P	6
Zhang et al. 2015 [83]	PD-1 PD-L1	China	Asian	NPC	139	113/26	H-score PD-1 > 0 PD-L1 > 35	IHC	I-IV	DFS	estimated	P	7
Zheng et al. 2017 [84]	PD-L1	China	Asian	NPC	85	63/22	Score > 2	IHC	I-IV	OS	estimated	P	6
Zhu et al. 2017 [38]	PD-L1	China	Asian	NPC	209	150/59	≥ 5%	IHC	I-IV	OS DFS	reported	P	7

PD-L1: programmed death ligand 1; PD-1: programmed death 1; IDO: indoleamine 2, 3-dioxygenase; M/F: male/female; NA: not available; OSCC: oral squamous cell carcinoma; HNSCC: head and neck squamous cell carcinoma; NPC: nasopharyngeal carcinoma; OPSCC: oropharyngeal squamous cell carcinoma; HPSCC: hypopharyngeal squamous cell carcinoma; SSCC: sinonasal squamous cell carcinoma; LSCC: laryngeal squamous cell carcinoma; cut-off value: the value that can be diagnosed as positive/high expression of an immune checkpoint molecule; AQUA: automated quantitative analysis; IHC: immunohistochemistry; IF: immunofluorescence; qRT-PCR: quantitative reverse transcription polymerase chain reaction; FACS: fluorescence-activated cell sorting; FISH: fluorescence in situ hybridization; OS: overall survival; DFS: disease-free survival; DMFS: distant metastases-free survival; DSS: disease-specific survival; PFS: progression-free survival; P: prospective; NOS: Newcastle–Ottawa Quality Assessment Scale.

### Methodological quality of the included studies

The quality of the included studies was generally high. Most of the studies mentioned the length of the follow-up period, and the majority provided adequate follow-up data for more than five years. Nevertheless, almost none of the prospective studies had an exposed cohort that sufficiently represented the general characteristics of the population in the community, as this factor was not considered in the study design. None of the studies were designed with adequate comparability of cohorts, due to their failure to match exposed and non-exposed individuals and/or adjust for confounders. Methods for handling missing data and intention-to-treat analysis were not adequately described in the majority of the studies.

### Immune checkpoint molecule expression and prognosis of HNC patients

**Figure 2:**
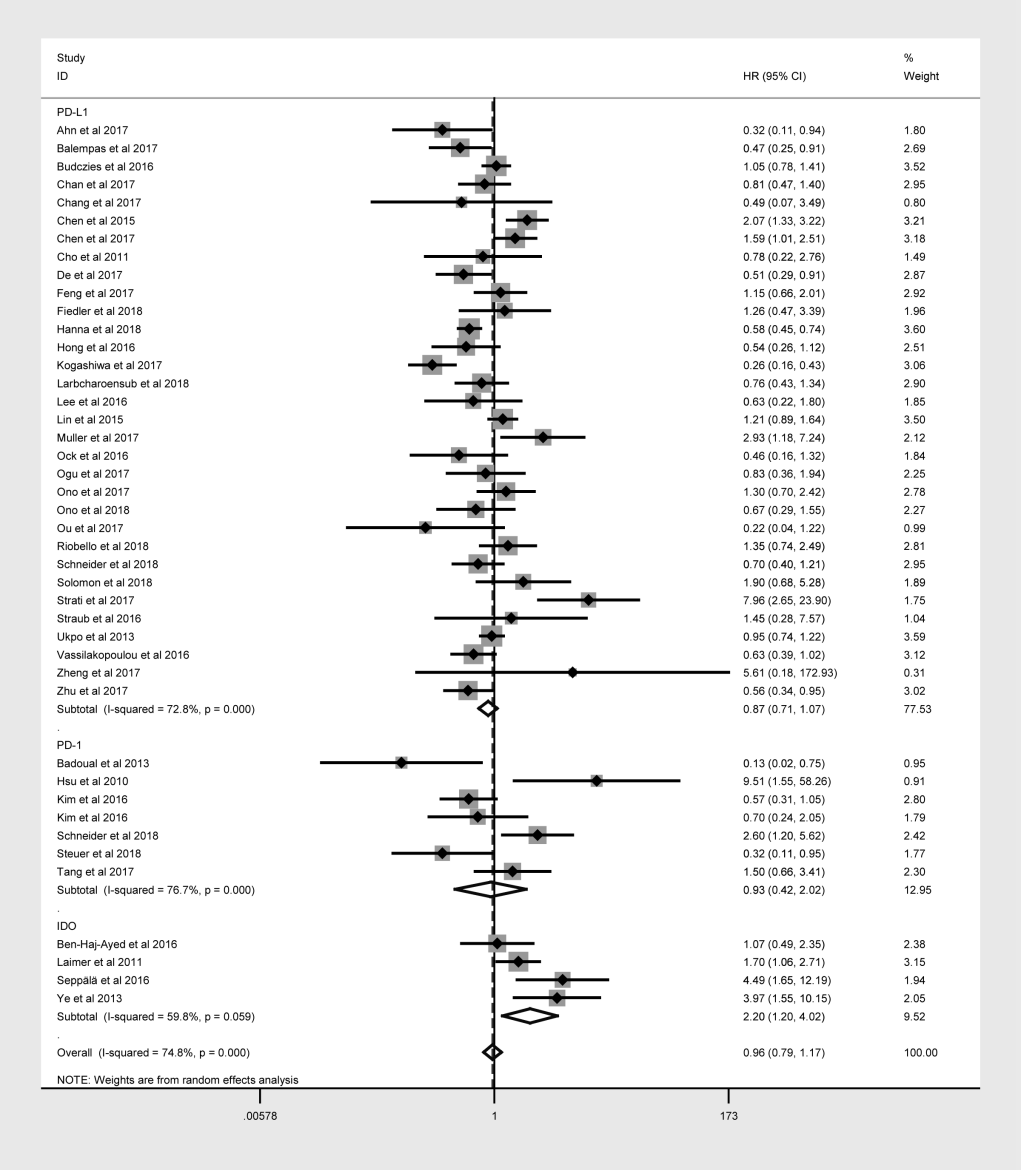
Overall forest plot of stratified analysis based on the type of molecule for the association of immune checkpoint molecules with OS.

Forty-three studies with 6225 patients reported the relationship between OS and at least one of the three immune checkpoint molecules in HNC. The expression of these molecules was detected mainly at the protein level, except for three studies that evaluated *PD-L1* mRNA levels. Overexpression was defined based on cut-off criteria that differed among the studies (as presented in Table 1). When the data for all three immune checkpoint molecules were pooled, there was no significant relationship between the overexpression of these molecules and OS (hazard ratio [HR] = 0.964; 95% confidence interval [CI]: 0.791-1.175, *P* = 0.714; Table 2), and there was obvious overall heterogeneity (I^2^ = 74.8%, *P*_h_ < 0.001; Figure 2). Similar results were obtained for DFS, PFS, DSS and DMFS.

**Table 2:** Results of the meta-analysis on the prognostic effects of immune checkpoint molecules in HNC patients.

	**Variable**	**Study no.**	**Sample size**	**HR (95% CI)**	***P* value**	**Heterogeneity**	
						**I^2^**	***P* value**
OS	Overall	43	6225	0.964 (0.791-1.175)	0.714	74.8%	<0.001
	**Immune checkpoint molecules**						
	PD-L1	32	4854	0.874 (0.711-1.073)	0.197	72.8%	<0.001
	PD-1	7	967	0.926 (0.424-2.025)	0.848	76.7%	<0.001
	IDO	4	404	2.197 (1.199-4.023)	0.011	59.8%	0.059
	**Ethnicity**						
	Asian	19	2938	0.923 (0.651-1.307)	0.650	77.1%	<0.001
	Caucasian	24	3287	0.995 (0.779-1.270)	0.965	73.8%	<0.001
	**Tumor location**						
	OSCC	13	1477	0.879 (0.586-1.317)	0.532	85.0%	<0.001
	NPC	10	1008	0.862 (0.618-1.203)	0.383	33.7%	0.139
	OPSCC	4	592	0.878 (0.532-1.450)	0.611	47.1%	0.129
	HPSCC	1	83	1.300 (0.700-2.415)	0.407	-	-
	SSCC	1	53	1.355 (0.739-2.485)	0.326	-	-
	LSCC	2	447	1.517 (0.252-9.126)	0.649	91.4%	0.001
	**Sample size**						
	Large	14	3721	1.044 (0.803-1.356)	0.748	74.0%	<0.001
	Small	29	2504	0.915 (0.687-1.220)	0.546	74.3%	<0.001
DFS	Overall	19	2901	1.097 (0.733-1.642)	0.652	92.5%	<0.001
	**Inhibitory immune checkpoint molecules**						
	PD-L1	13	2010	0.874 (0.523-1.459)	0.606	94.1%	<0.001
	IDO	2	258	1.725 (0.611-4.869)	0.303	59.5%	0.116
	PD-1	4	633	1.931 (0.716-5.211)	0.194	87.5%	<0.001
	**Ethnicity**						
	Asian	8	1252	1.131 (0.506-2.533)	0.764	93.6%	<0.001
	Caucasian	11	1649	1.060 (0.760-1.479)	0.731	73.9%	<0.001
	**Tumor location**						
	OSCC	3	247	0.609 (0.208-1.788)	0.367	70.8%	0.033
	NPC	6	666	1.339 (0.581-3.085)	0.494	92.5%	<0.001
	SSCC	1	53	1.834 (0.955-3.522)	0.068	-	-
	OPSCC	1	181	1.090 (0.783-1.518)	0.610	-	-
	LSCC	2	447	1.282 (0.242-6.783)	0.770	85.9%	0.008
	**Sample size**						
	Large	6	1756	0.844 (0.595-1.198)	0.343	75.5%	<0.001
	Small	13	1145	1.225 (0.764-1.963)	0.399	88.9%	<0.001
PFS	Overall	6	545	0.996 (0.585-1.685)	0.989	68.5%	0.007
	**Inhibitory immune checkpoint molecules**						
	PD-L1	6	545	0.891 (0.565-1.404)	0.989	68.5%	0.007
	**Ethnicity**						
	Asian	3	233	0.846 (0.492-1.455)	0.744	48.3%	0.144
	Caucasian	3	312	1.218 (0.372-3.993)	0.546	82.7%	0.003
	**Tumor location**						
	NPC	2	227	0.762 (0.506-1.149)	0.195	0.0%	0.935
	OSCC	1	84	0.576 (0.308-1.076)	0.084	-	-
	HPSCC	1	83	1.350 (0.740-2.463)	0.328	-	-
	**Sample size**						
	Large	1	161	0.770 (0.480-1.235)	0.279	-	-
	Small	5	384	1.067 (0.536-2.125)	0.853	73.0%	0.005
DSS	Overall	5	699	0.779 (0.330-1.839)	0.569	84.7%	<0.001
DMFS	Overall	3	361	0.599 (0.346-1.035)	0.066	0.0%	0.604

### Subgroup analyses

Subgroup analyses stratified according to the immune checkpoint molecule, patient ethnicity, tumor location and sample size were performed to detect potential sources of heterogeneity. In the stratification based on the immune checkpoint molecule (Figure 2), poorer OS was consistently found in patients with higher levels of IDO (Table 2), correlating with a poorer prognosis (HR = 2.197, 95% CI: 1.199-4.023, *P* = 0.011, Figure 2). However, no obvious trend in DFS was found according to IDO expression (Table 2).

**Figure 3:**
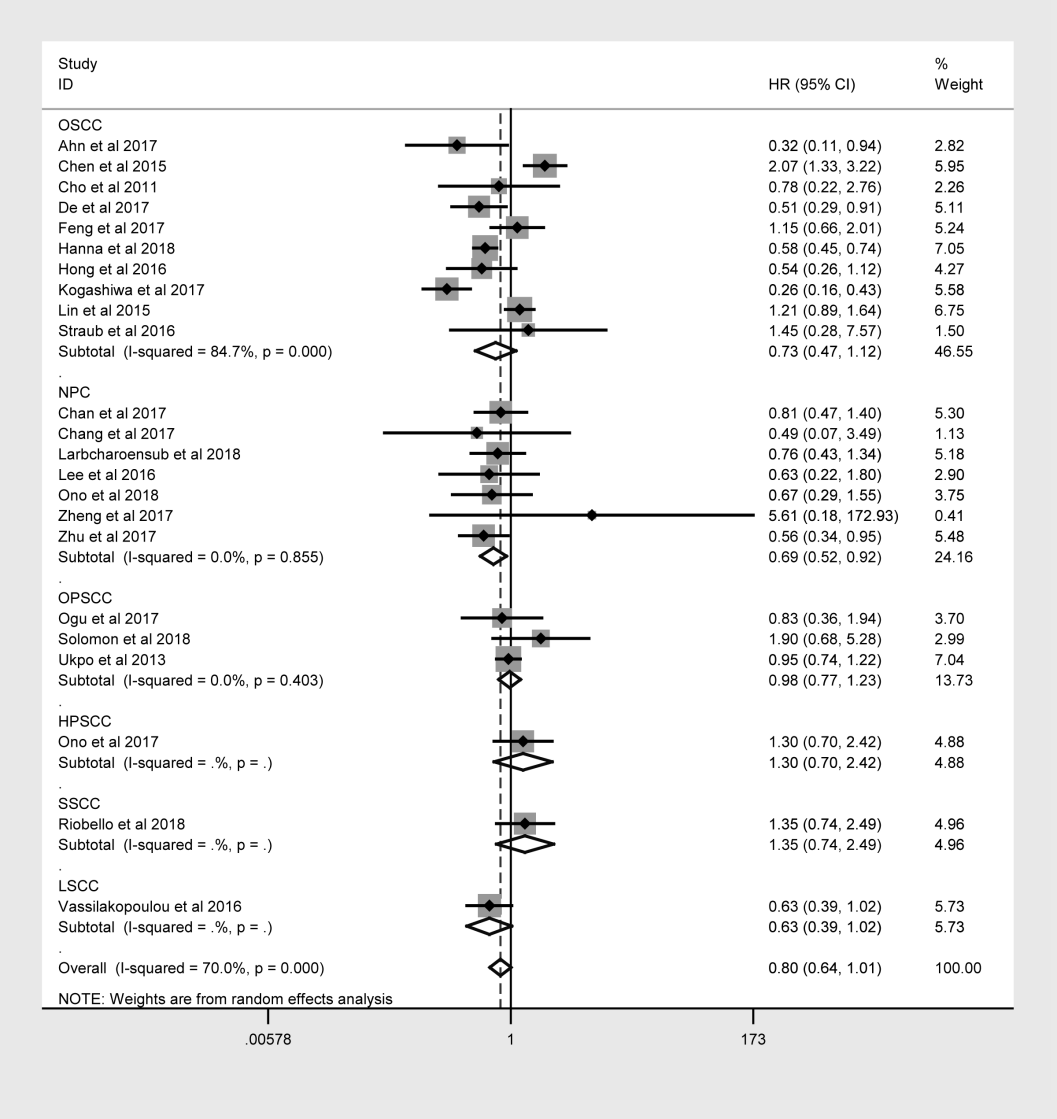
Overall forest plot of stratified analysis based on the tumor location for the association between PD-L1 and OS.

The same hierarchical strategy was used to evaluate the studies of PD-L1 (Table 3). Among the immune checkpoint molecules, PD-L1 was the focus of the largest percentage of studies, as 32 studies with 4854 patients reported the relationship between PD-L1 expression and OS (Figure 3). There was a possible trend for a better prognosis in patients overexpressing PD-L1 (HR = 0.874; 95% CI: 0.711-1.073, *P* = 0.197).

**Table 3:** Results of the meta-analysis on the prognostic effects of PD-L1 in HNC patients.

	**Variable**	**Study no.**	**Sample size**	**HR (95% CI)**	***P* value**	**Heterogeneity**	
						**I^2^**	***P* value**
OS	Overall	32	4854	0.874 (0.711-1.073)	0.197	72.8%	<0.001
	**Ethnicity**						
	Asian	14	2074	0.792 (0.537-1.168)	0.240	78%	<0.001
	Caucasian	18	2780	0.91 (0.716-1.158)	0.444	68.2%	<0.001
	**Tumor location**						
	OSCC	10	1198	0.726 (0.470-1.121)	0.148	84.7%	<0.001
	NPC	7	795	0.692 (0.523-0.915)	0.01	0.0%	0.855
	OPSCC	3	495	0.975 (0.771-1.234)	0.835	0.0%	0.403
	HPSCC	1	83	1.300 (0.700-2.415)	0.407	-	-
	SSCC	1	53	1.355 (0.739-2.485)	0.326	-	-
	LSCC	1	260	0.635 (0.393-1.025)	0.063	-	-
	**Sample size**						
	Large	12	3132	1.022 (0.790-1.321)	0.87	71.4%	<0.001
	Small	20	1722	0.77 (0.575-1.031)	0.08	66.6%	<0.001
DFS	Overall	13	2011	0.874 (0.523-1.465)	0.607	93.9%	<0.001
	**Ethnicity**						
	Asian	5	617	0.824 (0.290-2.338)	0.716	94.2%	<0.001
	Caucasian	8	1394	0.883 (0.638-1.221)	0.451	62.4%	0.009
	**Tumor location**						
	OSCC	3	247	0.610 (0.208-1.793)	0.369	70.5%	0.034
	NPC	4	549	1.042 (0.349-3.111)	0.941	94.9%	<0.001
	SSCC	1	53	1.834 (0.955-3.522)	0.068	-	-
	OPSCC	1	181	1.090 (0.783-1.518)	0.610	-	-
	LSCC	1	260	0.591 (0.350-0.997)	0.048	-	-
	**Sample size**						
	Large	4	1167	0.829 (0.597-1.151)	0.263	57.5%	0.07
	Small	9	844	0.900 (0.454-1.785)	0.762	91.7%	<0.001
PFS	Overall	7	630	0.996 (0.632-1.569)	0.986	62.1%	0.015
	**Ethnicity**						
	Asian	4	318	0.879 (0.585-1.321)	0.534	24.2%	0.266
	Caucasian	3	312	1.219 (0.372-3.997)	0.744	82.6%	0.003
	**Tumor location**						
	OSCC	2	169	0.706 (0.416-1.197)	0.196	7.8%	0.298
	HPSCC	1	83	1.350 (0.737-2.473)	0.331	-	-
	NPC	2	227	0.762 (0.503-1.154)	0.200	0.0%	0.935
	**Sample size**						
	Large	1	161	0.770 (0.476-1.246)	0.287	-	<0.001
	Small	6	469	1.058 (0.600-1.876)	0.845	66.2%	0.011
DSS	Overall	5	699	0.779 (0.330-1.839)	0.569	84.7%	<0.001
DMFS	Overall	3	361	0.599 (0.346-1.035)	0.066	0.0%	0.604

In nasopharyngeal carcinoma (NPC) patients, the OS was better for those expressing higher levels of PD-L1 (HR = 0.692, 95% CI: 0.523-0.915, *P* = 0.010). However, no obvious trend in DFS, PFS, DSS or DMFS was found according to PD-L1 expression. In laryngeal squamous cell carcinoma patients, higher PD-L1 expression was associated with better DFS (HR = 0.591, 95% CI: 0.350-0.997, *P* = 0.048).

### Sensitivity analysis and publication bias

**Figure 4:**
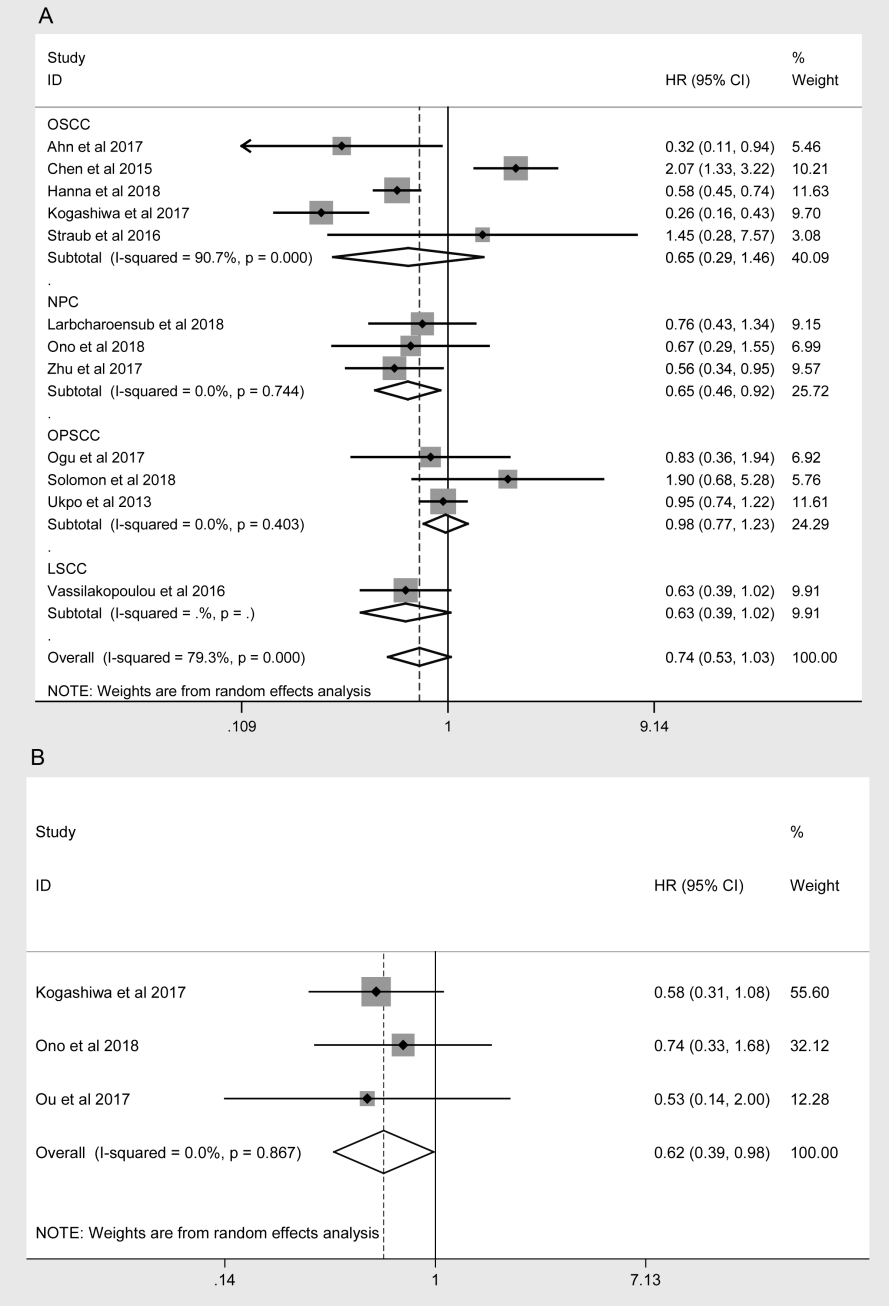
**Overall forest plots of sensitivity analysis.** (**A**) Stratified analysis based on the tumor location for the association between PD-L1 and OS. (**B**) Overall forest plots of sensitivity analysis for the association between PD-L1 and PFS.

A sensitivity analysis of the association between the expression of PD-L1 and the prognosis of HNC patients was performed for high-quality studies (NOS score ≥ 7, Table 4). The overall HRs and 95% CIs followed the same trends as those in the previous analysis. Higher levels of PD-L1 exhibited a trend of correlation with better OS (HR = 0.754, 95% CI: 0.568-1.002, *P* = 0.051, Figure 4A) and were associated with better PFS (HR = 0.618, 95% CI: 0.388-0.985, *P* = 0.043, Figure 4B) in the high-quality studies. As in the previous analysis, the OS of NPC patients was better in the high-PD-L1 group (HR = 0.649, 95% CI: 0.458-0.920, *P* = 0.015, Figure 4A). The heterogeneity among the studies decreased slightly for OS, but it remained statistically significant (I^2^ = 76.6%, *P*_h_ < 0.001; Table 4). In addition, subgroup analyses revealed that higher PD-L1 levels were associated with better OS in Caucasian patients (HR = 0.742, 95% CI: 0.578-0.954, *P* = 0.020) and in studies with small sample sizes (HR = 0.582, 95% CI: 0.426-0.796, *P* < 0.001, Table 4).

**Table 4:** Sensitivity analysis results for high-quality studies on the prognostic effects of PD-L1 in HNC patients.

	**Variable**	**Study no.**		**Sample size**	**HR (95% CI)**	***P* value**		**Heterogeneity**	
								**I^2^**	***P* value**
OS	Overall	17		2581	0.754 (0.568-1.002)	0.051		76.6%	<0.001
	**Ethnicity**								
	Asian	7		1255	0.720 (0.385-1.348)	0.305		88.1%	<0.001
	Caucasian	10		1326	0.742 (0.578-0.954)	0.020		46.1%	0.054
	**Tumor location**								
	OSCC	5		531	0.653 (0.292-1.462)	0.300		90.7%	<0.001
	NPC	3		389	0.649 (0.458-0.920)	0.015		0.0%	0.744
	OPSCC	3		495	0.975 (0.771-1.234)	0.835		0.0%	0.403
	LSCC	1		260	0.635 (0.393-1.025)	0.063		-	-
	**Sample size**								
	Large	7		1715	0.984 (0.659-1.468)	0.936		79.5%	<0.001
	Small	10		866	0.582 (0.426-0.796)	0.001		50.3%	0.034
DFS	Overall	7		1066	0.928 (0.618-1.392)	0.717		69.4%	0.003
	**Ethnicity**								
	Asian	3		416	0.809 (0.241-2.720)	0.732		85.8%	0.001
	Caucasian	4		650	0.938 (0.663-1.328)	0.719		43.6%	0.150
	**Tumor location**								
	OSCC		2	148	0.699 (0.114-4.263)		0.697	78.4%	0.032
	NPC		2	348	1.215 (0.288-5.133)		0.791	91.0%	0.001
	OPSCC		1	181	1.090 (0.783-1.518)		0.610	-	-
	LSCC		1	260	0.591 (0.351-0.996)		0.048	-	-
	**Sample size**								
	Large	3		650	0.753 (0.485-1.171)	0.208		66.8%	0.049
	Small	4		416	1.146 (0.536-2.450)	0.725		71.0%	0.016
PFS	Overall	3		188	0.618 (0.388-0.985)	0.043		0.0%	0.867

**Figure 5:**
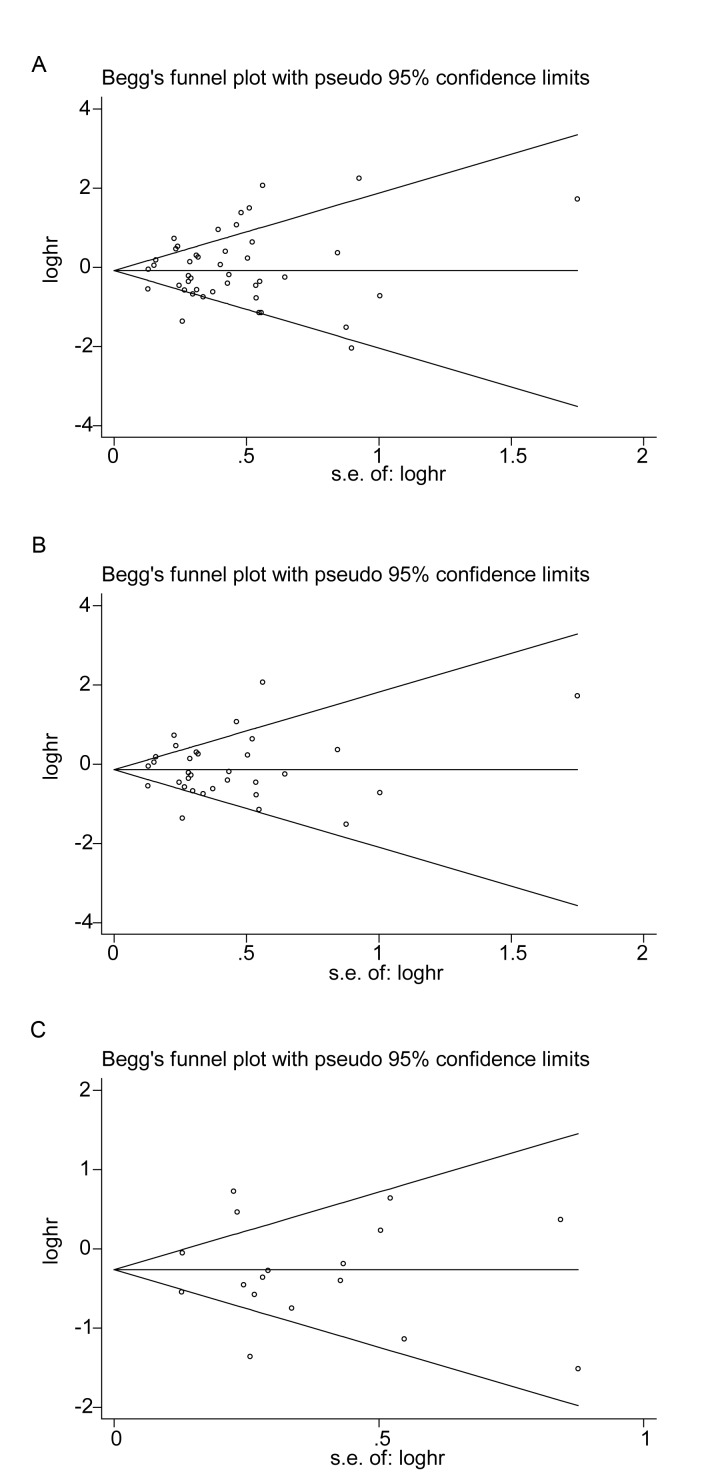
**Begg’s funnel plots of publication bias on the relationships between immune checkpoint molecules and OS** in all studies (**A**), PD-L1-associated studies (**B**) and high-quality studies on PD-L1 (**C**).

Funnel plots of OS were created for all the studies (Figure 5A), for the studies on PD-L1 (Figure 5B) and for the high-quality studies on PD-L1 (Figure 5C). For all three plots, the studies were distributed uniformly around the axis, manifesting no obvious publication bias (*P* = 0.509, 0.876 and 0.868 for all the studies, the studies on PD-L1 and the high-quality studies on PD-L1, respectively).

## DISCUSSION

As immune checkpoint molecules could be involved in the immune surveillance of tumor development and progression and the clearance of tumors [11], anti-immune-checkpoint drugs such as pembrolizumab [3,19], nivolumab [26,27] and ipilimumab [28] have been approved to treat melanoma, non-small cell lung cancer, renal cell carcinoma, prostate cancer and HNC. Recent studies have examined how immune checkpoint molecules, especially PD-L1, influence the prognosis of cancer patients, and a large number of updated reports have been published in the past two years. However, no consensus has been reached on the effects of immune checkpoint molecules on the prognosis of HNC.

This meta-analysis on the prognostic value of immune checkpoint molecules included 52 studies with a total of 7127 patients. The expression of immune checkpoint molecules was found to be a controversial prognostic factor for the OS, DFS, PFS, DSS and DMFS of HNC patients. Although the current view is that immune checkpoint molecules may be important predictors of a poor prognosis in HNC [17–19,22,29–31], our subgroup analysis stratified according to the immune checkpoint molecule revealed that different molecules had different associations with the patient prognosis. Thus, our results require careful attention.

Higher IDO expression was associated with a poorer prognosis for HNC patients in our study. Similarly, high IDO expression has been reported to correlate with a poor prognosis in patients with melanoma, breast cancer and colon cancer [32–34]. However, in our study, higher expression of PD-L1 tended to be associated with better OS. Kogashiwa et al. [35] found that higher expression of PD-L1 was associated with a higher number of CD8+ tumor-infiltrating lymphocytes, leading to better OS for HNC patients. Balermapas et al. and Hanna et al. [36,37] also reported higher levels of tumor-infiltrating lymphocytes in HNC patients expressing higher levels of PD-L1, which could explain the improved OS of these patients.

As PD-L1 attracted the most attention of the included immune checkpoint molecules, and a large number of updated studies reported the relationship between high levels of PD-L1 and the prognosis of HNC in 2017 and 2018, we considered it important to conduct a further meta-analysis solely on this molecule. We found that higher PD-L1 expression was associated with better OS in NPC patients, although for HNC overall there was only a positive trend, rather than a concrete link (Figure 3). A sensitivity analysis revealed the same trends in OS. In addition, higher PD-L1 expression was found to correlate with better PFS. The results of the sensitivity analysis may be more dependable than the former results, as all the included studies were of high quality. Furthermore, the same relationship between PD-L1 expression and OS was confirmed in Caucasian subjects, NPC patients and studies with small sample sizes.

Tumors can develop adaptive immune resistance, which is one of the two mechanisms regulating tumor PD-L1 expression (the second being intrinsic immune resistance) [38]. While the intrinsic mechanism leads to PD-L1 expression after oncogenic mutation [39], the adaptive mechanism causes tumor cells to express PD-L1 after they have been stimulated by interferon gamma secreted by CD8+ T cells [40,41]. Therefore, tumor-membranous PD-L1 levels could partly reflect the amount of tumor-infiltrating lymphocytes, especially cytotoxic T cells, accounting to some extent for the better survival of patients with higher PD-L1 levels.

There are several limitations to this meta-analysis. Firstly, the overall heterogeneity was high, so random effects models were required for the analysis, and there was less sensitivity to detect significant differences. Secondly, all the included studies were prospective, and the majority of studies did not have adequate random sequences or comparable cohorts, increasing the risk of bias. Thus, the quality of the included studies was not perfect. Lastly, the study populations were all of Asian or Caucasian ethnicity, which may have caused a population selection bias.

Our meta-analysis indicated that different immune checkpoint molecules correlated with different prognoses in HNC patients: higher IDO expression predicted a poorer prognosis, while higher PD-L1 expression was associated with a better prognosis. Furthermore, our study revealed that higher expression of PD-L1 was associated with significantly better OS in Caucasian subjects, NPC patients and studies with small sample sizes. In summary, our study suggested that the immune checkpoint molecules IDO and PD-L1 have potential prognostic value and applicability to immune therapy for HNC.

## METHODS

### Literature-search strategy

This literature search was performed on August 10, 2018 without any restrictions in region, publication type, journal or language. The databases of PubMed, Embase and the Cochrane Library were thoroughly searched with the following strategy: ((((((((((((((((((((((((((head and neck cancer [Title/Abstract]) OR head and neck squamous cell carcinoma[Title/Abstract]) OR head and neck neoplasm$[Title/Abstract]) OR HNSCC[Title/Abstract]) OR SCCHN[Title/Abstract]) OR HNC[Title /Abstract]) OR mouth neoplasms[Title /Abstract]) OR cancers of mouth[Title/Abstract]) OR oral[Title/Abstract]) OR laryn*[Title/Abstract]) OR pharyn*[Title/Abstract]) OR tongue[Title/Abstract]) OR oropharyn*[Title/Abstract]) OR nasopharyn*[Title/Abstract]) OR hypopharyn*[Title/Abstract]) OR trachea[Title/Abstract]) OR laryngopharyn*[Title /Abstract]) OR cervical tracheal[Title/Abstract]) OR cervical esophagus[Title/Abstract]) OR lip[Title /Abstract])) OR sinonasal[Title/Abstract]) OR head and neck cutaneous squamous cell carcinoma[Title/Abstract]) OR squamous cell carcinoma of the oral cavity[Title/Abstract]) OR salivary gland carcinoma[Title/Abstract]) OR SGC[Title/Abstract]) AND (((((((((((((((((((((((((b7-h3[Title/Abstract]) OR cd276[Title/Abstract]) OR b7-h4[Title/Abstract]) OR vtcn1[Title/Abstract]) OR btla[Title/Abstract]) OR b and t lymphocyte attenuator) OR cd272[Title/Abstract]) OR ctla-4[Title/Abstract]) OR cytotoxic t-lymphocyte-associated protein 4[Title/Abstract]) OR cd152[Title /Abstract]) OR ido[Title/Abstract]) OR indoleamine 2,3-dioxygenase[Title/Abstract]) OR kir[Title/Abstract]) OR killer-cell immunoglobulin-like receptor[Title/Abstract]) OR lag3[Title/Abstract]) OR lymphocyte activation gene-3[Title/Abstract]) OR pd-1[Title/Abstract]) OR programmed death 1 receptor[Title/Abstract]) OR pd-l1[Title/Abstract]) OR programmed death ligand 1[Title/Abstract]) OR pd-l2[Title/Abstract]) OR tim-3[Title/Abstract]) OR t-cell immunoglobulin domain and mucin domain 3[Title/Abstract]) OR vista[Title/Abstract]) OR v-domain ig suppressor of t cell activation[Title/Abstract]) OR b7-h1[Title/Abstract]). Two reviewers (Y.Q.J. and B.Y.) inspected all candidate articles independently. Discrepancies were resolved by discussion with the senior authors (Z.W. and B.C.).

### Inclusion and exclusion criteria

The available prospective comparative studies (cohort studies) were included in this study based on their conformance to the following inclusion criteria: 1) the association of immune checkpoint marker expression with OS/DFS/PFS/DSS/DMFS in HNC was reported; 2) the diagnosis of HNC was made based on pathological examination; 3) HRs and 95% CIs were provided or could be estimated from the text; 4) only the more recent or complete article was selected when multiple reports described the same population, to avoid the duplicate inclusion of data; and 5) articles were published as original research.

The exclusion criteria were: 1) reviews, meeting abstracts, letters; 2) animal model studies; 3) sample size < 30 patients; 4) insufficient data to estimate the HR and 95% CI; 5) the main type of tumor was not SCC; 6) the number of studies on a single molecule was less than three; and 7) the study design was not prospective.

### Data extraction and quality assessment

Two reviewers (Y.Q.J. and B.Y.) extracted the following information independently from the included studies: author, year of publication, study country or region, sample ethnicity, tumor location, follow-up period, sample size, gender, cut-off values of immune checkpoint molecules, detection method, TNM stage, and survival data such as OS, DFS, PFS, DSS and DMFS. The HR and 95% CI were either reported or calculated from the *P* value or Kaplan-Meier survival curve [42,43]. Disagreements were resolved by a senior reviewer (Z.W.).

Two reviewers (L.L.W. and W.X.M.) independently assessed the quality of the included studies by the NOS. A score of 0–9 was given to each study, and studies with NOS scores ≥ 7 were defined as high-quality. Consensus was reached by discussion with senior reviewers (B.C. and Z.W.) when there were inconsistent results. Importantly, the procedure of assessing the quality of the studies was blinded to the reviewers who extracted the data (Y.Q.J. and B.Y.).

### Statistical analysis

This meta-analysis was performed in accordance with recommendations from the Cochrane Collaboration and the Quality of Reporting of Meta-analyses guidelines [44,45]. The HR was used as a summary statistic for censored outcomes (OS, DFS, PFS, DSS and DMFS). HRs > 1 represented a poor prognosis in HNC.

Heterogeneity among the primary studies was evaluated by Cochrane’s Q statistic and the I^2^ statistic. A *P* value < 0.10 in Cochrane’s Q test or an I^2^ value > 50% indicates substantial heterogeneity among studies, so a random effects model was used to calculate the pooled HR and 95% CI in such cases. Otherwise, a fixed effects model was applied.

We used the mean sample size as the boundary between studies with large and small sample sizes. Subgroup analyses were carried out according to the immune checkpoint molecule, ethnicity, sample size and tumor location. Sensitivity analysis was applied to high-quality studies (NOS ≥ 7). Begg’s funnel plots were used to assess publication bias. All statistical analyses were conducted with STATA 12.0 statistical software (Stata Corporation, College Station, TX, USA). A two-tailed *P* value < 0.05 was considered statistically significant.

## SUPPLEMENTARY MATERIAL

Supplementary Table 1
