# Supplementary material for: Prognostic value of immune checkpoint molecules in head and neck cancer: a meta-analysis
Source: Aging (Albany NY). 2019 Jan 22;11(2):501–22. doi: 10.18632/aging.101756 (PMC6366990; doi:10.18632/aging.101756)
Supplement: Supplementary Table 1 [file aging-11-101756-s001.docx]

**Supplementary Table 1. Risk of bias in prospective studies based on the modified Newcastle-Ottawa Scale.**

| **Study** | ***SELECTION*** | | | | | ***COMPARABILITY*** | ***OUTCOME*** | | | ***Quality Score*** |
| --- | --- | --- | --- | --- | --- | --- | --- | --- | --- | --- |
|  | **Representativeness of the Exposed Cohort** | **Selection of the Non-Exposed Cohort** | **Ascertainment of Exposure** | **Demonstration That Outcome of Interest Was Not Present at Start of Study** | | **Comparability of Cohorts on the Basis of the Design or Analysis** | **Assessment of Outcome** | **Long Enough Follow-Up for Outcomes to Occur** | **Adequacy of Follow-Up of Cohorts** |  |
| Ahn et al. 2017 | 0 | 1 | 1 | | 1 | 1 | 1 | 1 | 1 | 7 |
| Badoual et al. 2013 | 0 | 1 | 1 | | 1 | 1 | 0 | 1 | 1 | 6 |
| Balempas et al. 2017 | 0 | 1 | 1 | | 1 | 1 | 1 | 1 | 1 | 7 |
| Ben-Haj-Ayed et al. 2016 | 0 | 1 | 1 | | 1 | 1 | 1 | 1 | 1 | 7 |
| Birtalan et al. 2017 | 0 | 1 | 1 | | 1 | 1 | 1 | 1 | 0 | 6 |
| Budczies et al. 2016 | 0 | 1 | 1 | | 1 | 1 | 1 | 0 | 0 | 5 |
| Chan et al. 2017 | 0 | 1 | 1 | | 1 | 1 | 1 | 0 | 1 | 6 |
| Chang et al. 2017 | 0 | 1 | 1 | | 1 | 1 | 1 | 0 | 0 | 5 |
| Chen et al. 2015 | 0 | 1 | 1 | | 1 | 1 | 1 | 1 | 1 | 7 |
| Chen et al. 2017 | 0 | 1 | 1 | | 1 | 1 | 1 | 1 | 1 | 7 |
| Cho et al. 2011 | 0 | 1 | 1 | | 1 | 1 | 1 | 1 | 0 | 6 |
| De et al. 2017 | 0 | 1 | 1 | | 1 | 1 | 1 | 0 | 1 | 6 |
| Fang et al. 2014 | 0 | 1 | 1 | | 1 | 1 | 1 | 0 | 1 | 6 |
| Feng et al. 2017 | 1 | 1 | 1 | | 1 | 1 | 1 | 1 | 0 | 6 |
| Fiedler et al. 2018 | 0 | 1 | 1 | | 1 | 1 | 1 | 1 | 1 | 7 |
| Hanna et al. 2018 | 0 | 1 | 1 | | 1 | 1 | 1 | 1 | 1 | 7 |
| Hong et al. 2016 | 0 | 1 | 1 | | 1 | 1 | 1 | 0 | 1 | 6 |
| Hsu et al. 2010 | 0 | 1 | 1 | | 1 | 1 | 0 | 0 | 0 | 4 |
| Kansy et al. 2017 | 0 | 1 | 1 | | 1 | 1 | 0 | 1 | 1 | 6 |
| Kim et al. 2016 | 0 | 1 | 1 | | 1 | 1 | 1 | 1 | 0 | 6 |
| Kim et al. 2016 | 0 | 1 | 1 | | 1 | 1 | 1 | 1 | 1 | 7 |
| Kogashiwa et al. 2017 | 0 | 1 | 1 | | 1 | 1 | 1 | 1 | 1 | 7 |
| Laimer et al. 2011 | 0 | 1 | 1 | | 1 | 1 | 1 | 1 | 1 | 7 |
| Larbcharoensub et al. 2018 | 0 | 1 | 1 | | 1 | 1 | 1 | 1 | 1 | 7 |
| Lee et al. 2016 | 0 | 1 | 1 | | 1 | 1 | 0 | 1 | 0 | 5 |
| Li et al. 2017 | 0 | 1 | 1 | | 1 | 1 | 0 | 1 | 0 | 5 |
| Lin et al. 2015 | 0 | 1 | 1 | | 1 | 1 | 0 | 1 | 1 | 6 |
| Muller et al. 2017 | 0 | 1 | 1 | | 1 | 1 | 1 | 0 | 1 | 6 |
| Ock et al. 2016 | 0 | 1 | 1 | | 1 | 1 | 1 | 0 | 1 | 6 |
| Oguejiofor et al. 2017 | 0 | 1 | 1 | | 1 | 1 | 1 | 1 | 1 | 7 |
| Oliveira-Costa et al. 2015 | 0 | 1 | 1 | | 1 | 0 | 1 | 1 | 1 | 6 |
| Ono et al. 2017 | 0 | 1 | 1 | | 1 | 1 | 1 | 1 | 1 | 6 |
| Ono et al. 2018 | 0 | 1 | 1 | | 1 | 1 | 1 | 1 | 1 | 7 |
| Ou et al. 2017 | 0 | 1 | 1 | | 1 | 1 | 1 | 1 | 1 | 7 |
| Qu et al. 2017 | 0 | 1 | 1 | | 1 | 1 | 1 | 0 | 1 | 6 |
| Riobello et al. 2018 | 0 | 1 | 1 | | 1 | 1 | 1 | 0 | 1 | 5 |
| Roper et al. 2017 | 0 | 1 | 1 | | 0 | 1 | 1 | 1 | 1 | 6 |
| Satgunaseelan et al. 2016 | 0 | 1 | 1 | | 1 | 1 | 1 | 0 | 1 | 6 |
| Schneider et al. 2018 | 0 | 1 | 1 | | 1 | 1 | 1 | 1 | 1 | 7 |
| Seppälä et al. 2016 | 0 | 1 | 1 | | 1 | 0 | 1 | 1 | 1 | 6 |
| Solomon et al. 2018 | 0 | 1 | 1 | | 1 | 1 | 1 | 1 | 1 | 7 |
| Steuer et al. 2018 | 0 | 1 | 1 | | 1 | 1 | 1 | 1 | 1 | 7 |
| Strati et al. 2017 | 0 | 1 | 1 | | 1 | 1 | 1 | 0 | 0 | 5 |
| Straub et al. 2016 | 0 | 1 | 1 | | 1 | 1 | 1 | 1 | 1 | 7 |
| Tang et al. 2017 | 0 | 1 | 1 | | 1 | 1 | 1 | 1 | 0 | 6 |
| Ukpo et al. 2013 | 0 | 1 | 1 | | 1 | 1 | 1 | 1 | 1 | 7 |
| Vassilakopoulou et al. 2015 | 0 | 1 | 1 | | 1 | 1 | 1 | 1 | 1 | 7 |
| Ye et al. 2012 | 0 | 1 | 1 | | 1 | 1 | 1 | 0 | 1 | 6 |
| Zhang et al. 2015 | 0 | 1 | 1 | | 1 | 1 | 1 | 1 | 1 | 7 |
| Zheng et al. 2017 | 0 | 1 | 1 | | 1 | 1 | 1 | 1 | 0 | 6 |
| Zhu et al. 2017 | 0 | 1 | 1 | | 1 | 1 | 1 | 1 | 1 | 7 |
